# POLYGENIC RISK SCORE FOR DIABETES PREDICTS POSTOPERATIVE ACUTE KIDNEY INJURY

**DOI:** 10.1093/geroni/igae098.0427

**Published:** 2024-12-31

**Authors:** Aleda Leis, Michael Mathis, Jennifer Smith, Bhramar Mukherjee, Carrie Karvonen-Gutierrez

**Affiliations:** University of Michigan, Ann Arbor, Michigan, United States; University of Michigan, Ann Arbor, Michigan, United States; University of Michigan, Ann Arbor, Michigan, United States; University of Michigan, Ann Arbor, Michigan, United States; University of Michigan, Ann Arbor, Michigan, United States

## Abstract

Large genetic studies often lack detailed phenotyping of complex conditions because doing so requires substantial resources and intensive testing. Given the high prevalence of these conditions, particularly among older adults, omission of such information may lead to biased results. This analysis examined the utility of a polygenic risk score for type 2 diabetes (PRST2DM) versus phenotypic diabetes status in predicting postoperative acute kidney injury (AKI). Data from 14,824 Michigan Genomics Initiative patients age ≥18 years who underwent non-cardiac, non-renal, non-urologic, and non-liver transplant surgical procedures were included. PRST2DM was generated using previously published weights (Mars 2020; pgscatalog.org); values were binned into deciles. AKI was defined using modified Kidney Disease–Improving Global Outcomes (KDIGO) criteria. Logistic regression models of phenotypic T2DM status and the PRST2DM were evaluated; both models were adjusted for a common set of preoperative and intraoperative factors. AKI prevalence was 5.0% (n=737). The odds of AKI were 52% higher among those with phenotypic T2DM versus those without T2DM. Similarly, those in the highest PRST2DM decile had 43% higher odds of AKI compared to those in the lowest decile. Model area under the curve values were largely consistent between the phenotype and PRS models. These findings support the utility of the PRST2DM to serve as a suitable proxy for phenotypic T2DM when a granular phenotype is not available, is subject to information bias, or may not yet have occurred. Such a use is novel and may allow for more dynamic analyses of outcomes in genetic cohorts with limited phenotyping.

